# Chemical Safety Assessment Using Read-Across: Assessing the Use of Novel Testing Methods to Strengthen the Evidence Base for Decision Making

**DOI:** 10.1289/ehp.1409342

**Published:** 2015-05-08

**Authors:** Elisabet Berggren, Patric Amcoff, Romualdo Benigni, Karen Blackburn, Edward Carney, Mark Cronin, Hubert Deluyker, Francoise Gautier, Richard S. Judson, Georges E.N. Kass, Detlef Keller, Derek Knight, Werner Lilienblum, Catherine Mahony, Ivan Rusyn, Terry Schultz, Michael Schwarz, Gerrit Schüürmann, Andrew White, Julien Burton, Alfonso M. Lostia, Sharon Munn, Andrew Worth

**Affiliations:** 1 Joint Research Centre, European Commission, Ispra, Italy; 2 Cosmetics Europe, Brussels, Belgium; 3 OECD (Organisation for Economic Co-operation and Development), Paris, France; 4 Procter & Gamble, Cincinnati, Ohio, USA; 5 The Dow Chemical Company, Midland, Michigan, USA; 6 Liverpool John Moores University, Liverpool, United Kingdom; 7 EFSA (European Food Safety Authority), Parma, Italy; 8 L’Oréal, Asnières-sur-Seine, France; 9 U.S. Environmental Protection Agency, Washington, DC, USA; 10 Henkel AG & Co, Düsseldorf, Germany; 11 ECHA (European Chemicals Agency), Helsinki, Finland; 12 SCCS (Scientific Committee on Consumer Safety), Luxembourg; 13 Procter & Gamble, Egham, UK; 14 Texas A&M University, College Station, Texas, USA; 15 The University of Tennessee, Knoxville, Tennessee, USA; 16 Tübingen University, Tübingen, Germany; 17 Helmholtz Centre for Environmental Research, Leipzig, Germany; 18 Institute for Organic Chemistry, Technical University Bergakademie Freiberg, Freiberg, Germany; 19 Unilever PLC, Milton Keynes, United Kingdom

## Abstract

**Background:**

Safety assessment for repeated dose toxicity is one of the largest challenges in the process to replace animal testing. This is also one of the proof of concept ambitions of SEURAT-1, the largest ever European Union research initiative on alternative testing, co-funded by the European Commission and Cosmetics Europe. This review is based on the discussion and outcome of a workshop organized on initiative of the SEURAT-1 consortium joined by a group of international experts with complementary knowledge to further develop traditional read-across and include new approach data.

**Objectives:**

The aim of the suggested strategy for chemical read-across is to show how a traditional read-across based on structural similarities between source and target substance can be strengthened with additional evidence from new approach data—for example, information from *in vitro* molecular screening, “-omics” assays and computational models—to reach regulatory acceptance.

**Methods:**

We identified four read-across scenarios that cover typical human health assessment situations. For each such decision context, we suggested several chemical groups as examples to prove when read-across between group members is possible, considering both chemical and biological similarities.

**Conclusions:**

We agreed to carry out the complete read-across exercise for at least one chemical category per read-across scenario in the context of SEURAT-1, and the results of this exercise will be completed and presented by the end of the research initiative in December 2015.

**Citation:**

Berggren E, Amcoff P, Benigni R, Blackburn K, Carney E, Cronin M, Deluyker H, Gautier F, Judson RS, Kass GE, Keller D, Knight D, Lilienblum W, Mahony C, Rusyn I, Schultz T, Schwarz M, Schüürmann G, White A, Burton J, Lostia AM, Munn S, Worth A. 2015. Chemical safety assessment using read-across: assessing the use of novel testing methods to strengthen the evidence base for decision making. Environ Health Perspect 123:1232–1240; http://dx.doi.org/10.1289/ehp.1409342

## Introduction

The research initiative “Safety Evaluation Ultimately Replacing Animal Testing (SEURAT)” (SEURAT-1 2011) was inspired by the considerations presented in the report of the U.S. National Research Council entitled *Toxicity Testing in the 21st Century: A Vision and a Strategy* (National Research Council 2007). The European Union (EU) policy to protect laboratory animals (EU 2010) and the need for a new systemic toxicity testing arising from the complete ban on animal testing for cosmetic ingredients within the EU (2009) provided additional impetus for this large-scale collaborative effort. SEURAT-1 is a first step to addressing the long term strategic target and is focusing on the replacement of current repeated dose systemic toxicity testing *in vivo* used for human safety assessment. Six research projects and a coordination action contribute to the initiative, and combine the research efforts of over 70 European universities, public research institutes, and companies. SEURAT-1 is a public–private partnership co-financed by the European Commission’s FP7 Health Programme (EC 2007) and Cosmetics Europe (2015).

The SEURAT-1 strategy (Whelan et al. 2011) adopts a toxicological mode-of-action (MoA) framework to describe how any substance may adversely affect human health (Ankley et al. 2010; Boobis et al. 2008; Krewski et al. 2010) and uses this knowledge to develop complementary theoretical, computational (*in silico*), and experimental (*in vitro*) models that predict quantitative points of departure, needed for safety assessment (Sturla et al. 2014). The research initiative aims to prove this concept on three levels (Whelan et al. 2012, 2013) by *a*) theoretical descriptions of adverse outcome pathways (AOP) based on existing knowledge, *b*) hypothesis-based testing strategies employing alternative *in vitro* and *in silico* methods with a clearly defined toxicity prediction goal, and *c*) applying existing information (e.g., physical chemical properties, *in vivo* animal data, human data) and, with selected data generated from alternative methods, achieving a regulatory-accepted safety assessment, based on MoA knowledge of the compound.

MoA describes a biological response to a specific chemical challenge, whereas an AOP is a conceptual construct describing biological activities, beginning with a molecular initial event (MIE), and progressing through different biological levels to an observable adverse effect in a population (Ankley et al. 2010; Boobis et al. 2008). AOP constructs primarily related to hepatotoxicity were developed within SEURAT-1 (Landesmann and Vinken 2013) and included in the inventories of the AOP Development Programme [Organisation for Economic Co-operation and Development (OECD) 2013a] and the AOP Wiki (OECD 2014b) initiatives of the OECD.

Predictive toxicity testing at the level 2 proof of concept is currently about to be finalized by the SEURAT-1 partners (Berggren 2014) and will be used in the level 3 case studies. The consortium will carry out two separate case studies for applied safety assessment: the *ab initio* and the read-across case study. The *ab initio* case study will use results from the SEURAT-1 methods to make a risk assessment for repeated dose toxicity predicting a no-effect level of a cosmetic ingredient, assuming a certain exposure scenario. The primary goal of the read-across case study is to increase confidence in read-across assessment by using data from alternative methods. This approach will use a no-effect level based on existing data for one substance and read it across to a similar substance, and the resulting safety assessment is expected to reach regulatory acceptable standards within the SEURAT-1 time frames. In contrast, the *ab initio* case study, which relies solely on data from alternative methods, is considered an initial step towards a new alternative risk assessment strategy. The Joint Research Centre, European Commission, organized a workshop with invited experts to define the read-across case study in Ispra, Italy, 29–30 April 2014, entitled “The read-across case study for safety assessment contributing to the SEURAT-1 proof of concept.” The outcome of the workshop is the basis for this review.

Traditional read-across is considered a nontesting method for filling data gaps which is based on an analogue or chemical category (van Leeuwen et al. 2009). There must be scientifically credible arguments to support the read-across, and the inherent uncertainties must be addressed as uncertainty factors as often applied in weight-of-evidence approaches (Cronin et al. 2013). The intention with the SEURAT-1 case study is to show how a traditional read-across based on structural similarities [European Chemicals Agency (ECHA) 2008, 2012, 2013a, 2013b] can be strengthened with additional new data from *in vitro* testing, as well as complementary *in silico* data, when the structural similarities would be judged as insufficient to reach the necessary regulatory standard—for example, for registration under the Regulation concerning the Registration, Evaluation, Authorisation and Restriction of Chemicals (REACH) (EU 2006). The challenge to meet regulatory acceptance by increasing the confidence in the read-across was also described in a recently published case study (Ball et al. 2014).

A large proportion of higher-tier toxicological studies in the REACH registration dossiers already apply read-across approaches. It was reported that 75% of all registration dossiers include read-across or categorization reasoning (ECHA 2014), and the highest rate of read-across was used for repeated dose toxicity. However, this does not mean that all read-across predictions satisfy the regulatory requirements. It rather shows that there is further need to improve the methodology on how to perform read-across and categorization to achieve the standard for regulatory acceptance.

In the read-across context, the source substance is a substance with available *in vivo* data, and the target substance is a similar substance without sufficient data. If the quality of the read-across case study should be to meet the regulatory standard of substance registration under REACH, the ultimate aim would be to read across the full findings of a 90-day repeated dose oral toxicity rat study from a source substance to a target substance, with a high degree of confidence in the predicted set of toxicological properties for the target substance. Within SEURAT-1, a conceptual framework is developed to combine data from *in vitro* methods and other evidence to predict the toxicological properties of a substance (White and Knight 2014). This conceptual framework can be used for the case study as a rational basis for supporting the scientific justification of read-across. Other frameworks were proposed using structural, reactivity, metabolic, and physicochemical similarity to evaluate the suitability of read-across analogues (Blackburn et al. 2011; Wu et al. 2010), and the intention here is to further extend such a framework to include also new approach data, for example, information from *in vitro* molecular screening, “-omics” assays and computational models.

In our case study, the read-across justification, first, will be based on mechanistic understanding in combination with toxicokinetic and toxicodynamic assessment; second, it will be strengthened by selected *in vitro* data. An advantage of using data from *in vitro* models is the opportunity of using human cells or human-derived cell lines, with higher human relevance than traditional *in vivo* animal data. We therefore suggest the availability of human data as an additional criterion for the chemical selection, instead of using the animal studies as a gold standard. In the case study we select primarily liver as the target organ, because there is a large focus on hepatotoxicity within the SEURAT-1 projects; relevant AOP knowledge has been gathered and many data will be available for well characterized liver-derived cells of human origin.

In this review we describe the preparatory step for the SEURAT-1 read-across exercise, setting up a strategy and identifying suitable scenarios and chemical categories for further investigation. The safety assessment based on this strategy will be executed within the time frames of SEURAT-1, and the final evaluation will be reported by the end of 2015.

## Methods

*Read-across scenarios.* Considering chemical similarities, we must assess different aspects to make the read-across scientifically justified, such as the chemical stability, possible formation of toxic metabolites, different active groups that might lead to similar or dissimilar behaviors, possible routes of exposure and concentrations at the target tissue, biotransformation (before reaching, or at, the target organ), or observable trends. To improve and standardize the read-across assessment, it is therefore useful to identify different scenarios into which it would be possible to allocate any category of substances or substance analogues. This can be accomplished in several ways, but we agreed to include the toxicokinetic fate of the substance, specifically, whether adverse effect is caused by the activity of the compound itself at the target organ or by its metabolites or reaction products. Further, we agreed to include toxicodynamic behavior of the substance by assessing similarities in mechanism of action or lack of biological activity.

Four different scenarios are proposed to cover the most relevant read-across decision contexts:

Chemical similarity of compounds that do not require (or do not undergo) metabolism to exert a potential adverse human health effect
Chemical similarity involving chemical’s metabolism (resulting in exposure to the same/similar proximal toxicant)
Chemicals with generally low or no toxicity
Distinguishing chemicals in a structurally similar category with variable toxicities based on MoA hypothesis.

The first decision context involves substances, with no metabolism or very slow metabolism, that reach the target organ and are not converted to toxic metabolites. For *in vivo* studies it is often unknown whether the toxic effect is caused by the parent compound or the metabolite(s). However, scenario I is based on similarity of parent compounds and arguments for the exclusion of metabolites.

Read-across in the context of scenario II is based on the similarity of metabolites, The compounds in this scenario may have less structural similarity than compounds in scenario I, but can be characterized by identical substructures that results in similar, or even identical, metabolites. Sufficient biological *in vitro* evidence might be difficult to find for this decision context, as many currently available *in vitro* models lack metabolism to the degree necessary to provide proof of main metabolites and detect the related effects. However, the HepaRG cells used for toxicity prediction within SEURAT-1 (Lostia 2014; Noor and Heinzle 2014) have metabolic activity, and may therefore provide data that can be used as a basis for risk assessment. Evidence on any expected differences in the toxicokinetics of the source and target substances is a regulatory requirement (ECHA 2013a).

For decision context III, low or no toxicity, the problem is to identify on what basis to read-across; for example, in an absence of toxicity, there would be no common mechanism of action between source and target compound. Therefore, negative read-across may require more information to achieve the same level of certainty as a positive read-across (ECHA 2013a). Scenario III is of high importance from a safety assessment aspect because many cosmetic ingredients fit into this scenario, as well as substances used in other consumer products. Therefore read-across approaches that would support a conclusion of low toxicity are needed to empower the use of new less toxic substances. We agreed that for this scenario it would be necessary to strengthen the biological evidence for read-across by using *in vitro* assays relevant across different mechanisms of action to provide a general profile of low or no adversities.

The decision context IV aims to establish a strong hypothesis-driven scenario based on mechanistic reasoning with structural analogues, including at least one molecule with markedly different potency. The challenge will then be to determine the mechanistic causal relationship explaining why a certain analogue or analogues will lead to adverse outcome and others not.

The workshop participants considered that the four scenarios would cover all possible read-across situations, and each scenario will be further investigated by selecting well-characterized chemical categories.

*Chemical selection.* The most critical issue in any read-across exercise is the justification of analogue(s) selection. To gain acceptance, it is essential to explain the basis for similarity between the target chemical and potential source chemicals in a robust and reliable manner (Cronin et al. 2013). It is also necessary to assess which types of uncertainties are related to the read-across prediction—for example, the number of analogues in the source set, the concordance with regard to the data, and the severity of the hazard (Blackburn and Stuard 2014).

Chemical categories or pairs of analogues. We decided to use chemical categories for the case study, rather than using pairs of individual chemicals, because data available for any source substance in a category may be used to fill information gaps for substances with missing data. However, when making the chemical selection for the case study scenarios, we required that at least one analogue in the category must be well studied, with high-quality animal and human *in vivo* data, to reduce uncertainty. This criterion would be met by choosing a category including at least one of the SEURAT-1 gold compounds (ToxBank 2014; Wiseman 2012). This is a set of reference compounds with rich database and known MoA for target organ systemic toxicity, selected by SEURAT-1 partners to test the methods developed within the projects. Chemicals can also be selected from ToxRefDB (U.S. EPA 2010) which includes hundreds of chemicals and thousands of *in vivo* animal toxicity studies, the RIFM (Research Institute for Fragrance Materials) Database (RIFM 2014) or assessments made by the Scientific Committee on Consumer Safety (SCCS) (EC 2013). In addition, the SEURAT-1 project COSMOS (COSMetics to Optimise Safety) (COSMOS 2011) has developed a database, COSMOSdb (COSMOS 2014), that includes a large number of toxicity studies with emphasis on substances used in cosmetics.

We agreed to choose categories for which toxicology studies are available for both target and source substances to facilitate the assessment of whether additional information actually is strengthening the read-across case.

Chemical and biological similarity. In this case study a chemical category is based primarily on chemical similarity and then supported by biological arguments, but it could be considered to select chemicals based on their biological similarity, because of a common MoA or data profile, with less degree of chemical similarity. Structurally dissimilar chemicals can have similar toxicological properties, and there might be reasons to start from biological similarities rather than being limited to the chemical structure thinking (Low et al. 2013), which is how read-across traditionally is made. A possibility could be to start either from the *in vitro* data profiles and then look for the structural similarities possibly causing the similar MoA. A best approach would be to keep an open mind and start either from chemical or biological similarity, and it should rather be a matter of fit for purpose based on the available information. The toxicity signature–driven approaches are still difficult due to the uncertainty in the relevance of the data derived from the novel *in vitro* toxicity studies. However, we suggest that for substances of complex, and frequently poorly characterized, chemical composition—such as petrochemicals and other mixtures of unknown or variable composition, complex reaction products, or biological materials—it is best to approach read-across from the perspective of a biological similarity signature. This is to avoid the inherent challenge of using chemical similarity as the main basis of read-across when there is uncertainty and variability in chemical composition.

It would be useful to identify closest neighbors both in a chemical and biological space for a more holistic view of the problem. Therefore it is necessary to progress and identify the biological space and further explore how to visualize the biological data. We suggest that the integrative chemical–biological read-across (CBRA) approach (Low et al. 2013) for chemical hazard classification can be used to illustrate chemical and biological similarities between analogues. The U.S. EPA interactive software ToxPi (http://comptox.unc.edu/toxpi.php) (Reif et al. 2010, 2013) is also providing a tool to illustrate the biological data within and across the categories. ToxREAD (http://www.toxgate.eu/) is a recently developed tool assisting in searching for similar chemicals considering both chemical and biological data from existing databases supporting a read-across assessment.

More efficient use of *in silico* tools. There are several potential approaches for assessing chemical structure similarity among target and source compounds. The frequently used Tanimoto index (Johnson and Maggiora 1990; Rogers and Tanimoto 1960) can be applied to compute similarity between structural fingerprints derived from one or more types of chemical descriptors; however, it can also be extended to cover mechanistic similarity derived from *in vitro* studies, because it relies on a comparison of two bit strings, in which the 1s and 0s represent the presence or absence of different features (e.g., absence or presence of a given biological effect). Additional criteria could be applied when defining chemical categories based on biologically relevant functional groups and chemical moieties, as well as understanding of potential metabolic divergence between analogues. We suggest using “atom centric fingerprinting” (Todeschini and Consonni 2009), as a molecular descriptor for examining structural similarity, because it does not rely on a pre-built (subjective) set of substructures. Other available *in silico* tools should also be used to better inform on the biological signature to supplement the examination of structural similarity.

We will identify more descriptors by further exploiting the OECD QSAR (quantitative structure–activity relationship) Toolbox (OECD 2013b). For example *in silico* predictors could be used to evaluate metabolic similarities to better identify the members of a chemical category. In the OECD QSAR Toolbox, usually a broad category is first defined (e.g., using chemical alerts for protein reactivity), and then the category can be refined adding more criteria. There is the need for mechanistic categorization to strengthen the confidence in similarity of members of a category, such as chemical alerts based on a specific mechanism, for example, DNA or protein binding.

*A new read-across strategy.* Our strategy is to divide the exercise in two steps. The first step will include building up the read-across case based on physico-chemical and molecular properties, substituents, functional groups, and extended structural fragments, two-dimensional molecular similarity (i.e., statistical similarity based on graph theory), structural alert for chemical–biological interaction, and AOP knowledge. Step 1 would then correspond to read-across without additional new approach data. Step 2 will add such data primarily from the ToxCast data from *in vitro* high throughput screening assays (Kleinstreuer et al. 2014; U.S. EPA 2014) and from application of the alternative methods developed within the SEURAT-1 initiative. Thus, read-across assessments will be conducted twice, first without any new approach data and then again integrating alternative data into the assessment and independently judging for robustness/confidence before and after the addition of the alternative data.

The *in vitro* data will need to be further complemented with toxicokinetic data for application in safety assessment. Further toxicokinetic evidence will be provided from the SEURAT-1 projects and is already available for 500–800 chemicals in ToxCast (Wetmore et al. 2012). The final goal is to evaluate whether the new approach data managed to strengthen the confidence in the read-across within a selected category. An added value will be to identify further *in vitro* data to be sought even if not achievable within the timelines of SEURAT-1.

A chemical category is chosen based on a clear hypothesis why the AOP would be possible to read-across in between source and target. Alternative data might be available only for certain key events, but might still provide confidence in that the substances trigger the same AOP. In addition, the number of analogues in a category must include one data rich source chemical with a known MoA and at least four target chemicals, preferably with available data that can be used to build up or confirm the read-across arguments. We considered that categories of 5–10 substances would be a workable size. At least one analogue should be hypothesized to be an outlier, a ringer, in the category—that is, expected to be a target compound for which the read-across from the source compound will be clearly not acceptable. The inclusion of an outlier raises the confidence in the read-across from the source to the other target compounds.

## Discussion

We applied the chemical selection criteria and agreed on chemical groups for each of the four read-across scenarios. To assess each chemical category, templates will be developed for recording and evaluating *a*) traditional toxicology data, *b*) alternative data, and *c*) robustness/confidence in read-across assessment (Schultz et al. 2015). For *b*) and *c*), common criteria are necessary to make it possible to compare the different read-across categories and understand whether the alternative data strengthened the confidence in reading across.

*Scenario I: Chemical similarity of compounds that do not require (or do not undergo) metabolism to exert a potential adverse human health effect.* Category I.I: Perfluoroalkyl acids (PFAAs). An example of a direct-acting toxicant with no metabolism would be perfluorooctanoic acid (PFOA). There are extensive animal and human *in vivo* data available on the potential “source” chemicals PFOA and perfluorooctane sulfonate (PFOS) (Nelson et al. 2010). Many alternatives have been synthesized and proposed for replacement, enabling both analogue and category types of read-across. The target compounds could be selected within the PFAAs that have different chain lengths. Chemical similarity among analogues is substantial and well defined. For the step 2 assessment there is available toxicogenetics (O’Shea et al. 2011) and ToxCast data (U.S. EPA 2014).

The toxicokinetics of these compounds, most well studied for PFOA, is very different between experimental animals and humans, and also between sexes in some species. Blood half-life of PFOA in humans is several years, whereas in all other species it varies from hours to weeks. The species differences in toxicokinetics are thought to be attributable to renal reabsorption (Wambaugh et al. 2013). MoA data are most extensive for liver effects, and there are more limited data on the mechanisms of endocrine-disruption effects. There is some evidence for peroxisome proliferator–activated receptor (PPAR) and other nuclear receptor (NR) activation; however, there is no evidence for metabolism, and it is the displacement of endogenous fatty acids from the transport proteins that may result in the disruption of PPAR pathways (Zhang et al. 2013).

**Figure 1:**
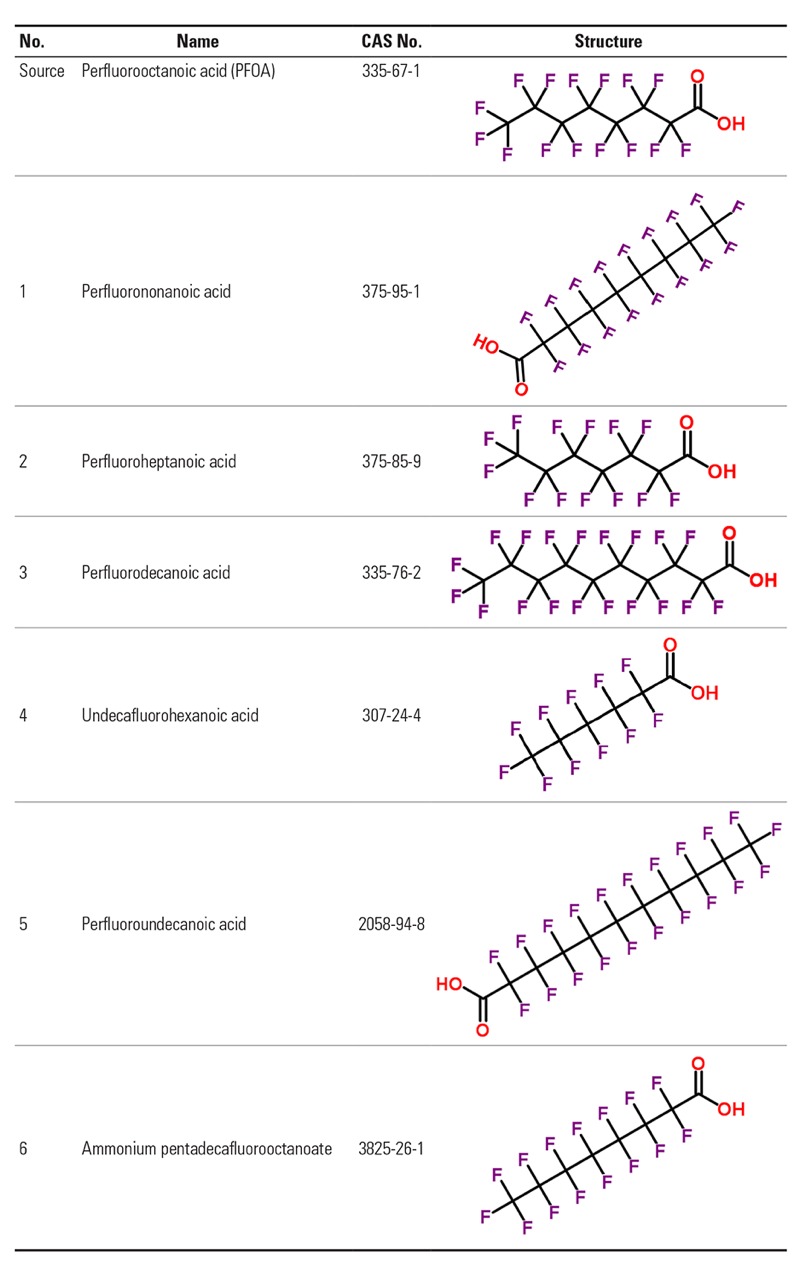
Chemicals suggested for Category I.I: Perfluoroalkyl acids (PFAAs), identified by names, CAS (Chemical Abstracts Service) numbers, and molecular structures. The substance indicated “source” is the most data-rich substance in the category with known MoA.

Potential category analogues for the PFAAs are illustrated in Figure 1. The hypothesis is that they are direct-acting toxicants with a similar MoA. We agreed to investigate this category further within the SEURAT-1 read-across study. *In vitro* data for step 2 will be collected primarily from ToxCast assays (U.S. EPA 2014). In addition, predictive computational tools developed within the SEURAT-1 project COSMOS will be applied.

Category I.II: Phthalates. Phthalates are a group of chemicals that form a number of proximal non-electrophilic metabolites that are ligands for PPARα and other NRs. Reproductive toxicity of phthalates is the greatest human health concern compared with other end points, for example, cancer (Rusyn and Corton 2012). Many alternatives have been synthesized and proposed as replacements, enabling both analogue and category types of read-across. Extensive *in vivo* data are available for several compounds in this category (U.S. EPA 2010), as are human data for at least one potential source chemical, diethylhexyl phthalate (DEHP), including toxicokinetics data [U.S. Agency for Toxic Substances and Disease Registry (ATSDR) 2002]. *In vitro* data are available from ToxCast assays (U.S. EPA 2014). However, we agreed not to include the phthalates in the SEURAT-1 case study because we considered that toxicity due to metabolites could not be excluded.

Category I.III: Pesticides. Several pesticide groups could fit into the first scenario. For some chemical classes of pesticides, metabolism in mammalian systems does not yield reactive intermediates. Often many alternatives have been synthesized and proposed as replacements, enabling both analogue and category types of read-across. There is a large source of *in vivo* animal data resulting from the many regulatory studies required and available in ToxRefDB (U.S. EPA 2010) or through the European Food Safety Authority (EFSA 2015). MoA data are usually extensive for the pesticidal property being a desired effect. ToxCast assay data are available for a vast number of pesticides (U.S. EPA 2014).

It would be necessary to further investigate whether a suitable category for scenario I could be identified among pesticide active ingredients. However, this is currently not intended to be followed up within the SEURAT-1 initiative.

*Scenario II: Chemical similarity involving metabolism (resulting in exposure to the same/similar toxicant).* In this scenario, the emphasis would be on facilitating read-across based on toxicokinetic properties. Metabolism simulators from the OECD QSAR Toolbox (OECD 2013b) could be used. If the metabolic profile looks the same between the source and target substances, this could be sufficient for reading across. In the second assessment step looking at *in vitro* data, it is essential to select *in vitro* systems with relevant and well-defined metabolic activity. It must be further evaluated to what degree the specificity and reactivity in metabolizing of the substance could be considered.

Category II.I: β-Unsaturated alcohols (allylic alcohols). The β-unsaturated alcohols undergo metabolic transformation to the corresponding β-unsaturated aldehyde via cytosolic alcohol dehydrogenase (ADH) (Bradbury and Christensen 1991). ADH is expressed in HepaRG cells, so allylic alcohols can be tested using methods developed and applied within SEURAT-1, where several partners are using HepaRG as a model system. Formation of the toxic metabolite acetaldehyde, which is the proximal toxicant, would subsequently lead to liver fibrosis through ROS (reactive oxygen species) formation in repeated dose *in vivo* studies (Landesmann et al. 2012). On the other hand, ADH is not expressed in HepG2 (Wilkening et al. 2003), which is why this cell line exposed to allylic alcohols can be used as negative control.

Also, data are available from ToxCast assays (U.S. EPA 2014) that can be used as a basis for the decision on further testing. The group with the greatest number of analogues in ToxCast (U.S. EPA 2014) was made by selecting allylic alcohols based on structural similarity only (e.g., using the Tanimoto index). However, *in vitro* data signatures varied substantially among compounds included in this group. This might be explained by the fact that all allylic alcohols are metabolized, but not to the same metabolite (Strubelt at al. 1999), which makes the group unique and specific. The use of cell lines that lack metabolic capacity might also produce dissimilar ToxCast data among compounds included in the group. The difficulty in interpreting the ToxCast data demonstrated the need for a well-structured hypothesis in choosing the compounds of the category.

**Figure 2:**
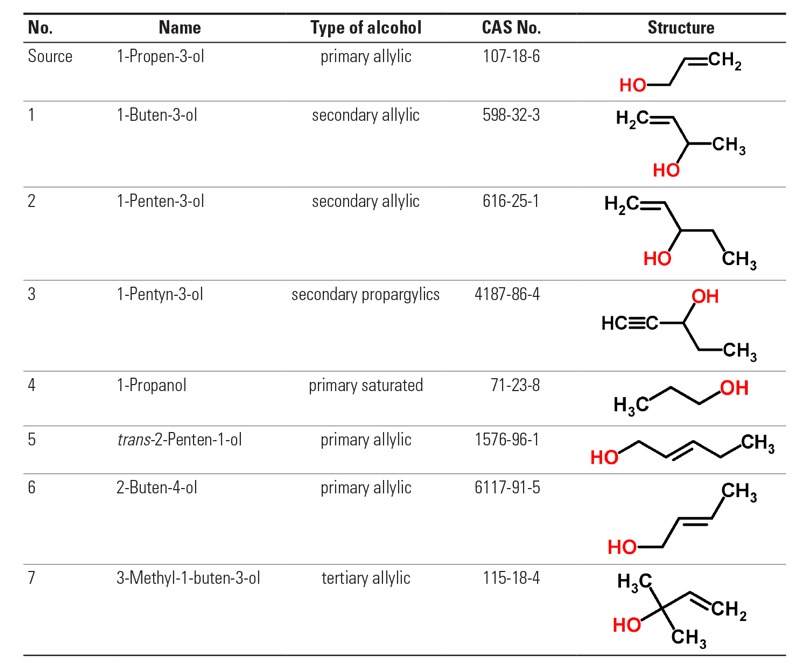
Chemicals suggested for Category II.I: β-unsaturated alcohols (allylic alcohols), identified by names, CAS numbers, and molecular structures. The substance indicated “source” is the most data-rich substance in the category with known MoA.

1-Propen-3-ol is a SEURAT-1 gold compound (Wiseman 2012; ToxBank 2014) with a rich *in vivo* database, so it is suggested as a suitable source chemical in this category. Possible target chemicals are listed in Figure 2.

Our hypothesis for this read-across case study is that β-unsaturated alcohols are indirect-acting toxicants with a similar MoA, where metabolism via ADH leads to necrosis and apoptosis and subsequent liver fibrosis (Landesmann et al. 2012). Thus, repeated dose toxicity for allyl alcohol can be read-across to the other β-unsaturated alcohols within the category that are metabolized to their corresponding α,β-unsaturated aldehyde via the same mechanism.

We predict *a priori* with high confidence that compounds 1–3 in Figure 2 elicit liver fibrosis as the source compound allylic alcohol. Chemical 4, 1-propanol, is the “ringer” chemical (a non-β-unsaturated alcohol) and is predicted to be an outlier to the category. Chemicals 5 and 6 are less likely to be members of the chemical category defined by the source compound allylic alcohol because the unsaturated group is not the terminal group in these cases. We assume that chemical 7, 3-methyl-1-buten-3-ol, is an outlier to the category because it lacks the ability to be metabolized to a corresponding β-unsaturated aldehyde via ADH.

We selected the category to be further investigated within the SEURAT-1 read-across study. *In vitro* data will be collected from SEURAT-1 assays, including the bioreactor models developed within the SEURAT-1 HeMiBio project (HeMiBio 2011) to predict fibrosis, for the step 2 read-across exercise.

Category II.II: Halogenated solvents. Another candidate category to be further evaluated under this scenario could be the halogenated solvents: chlorinated and fluorinated solvents. Substances such as trichloroethylene or tetrachloroethylene are extremely data rich due to the rigorous risk assessments made in the past (EC 2004, 2005; U.S. ATSDR 2014a, 2014b). Also, the kinetics would be possible to predict for these compounds. It was agreed that these solvents would be good examples because the chemical read-across was poor and would need strengthening arguments from metabolic and mechanistic data. However, we agreed not to progress with this category within the time frame of the SEURAT-1 projects.

Category II.III: Pharmaceuticals, such as acidic drugs or acetaminophen (Tylenol®, Paracetamol®) with analogues. The best knowledge on metabolites can be found for pharmaceuticals. A problem for this group of chemicals is that many of the data are not publicly available, which is why the category was not chosen to be carried forward in the SEURAT-1 case study. However, it is recognized that acidic drugs could be of particular interest due to their known UGT-mediated activation to acyl glucuronides (Davis and Hanumegowda 2009). They are also widely studied and, therefore, data rich. Acetaminophen is another example of an extremely data-rich substance that could be used as a source with a known mechanism through P450-mediated activation to electrophilic substance (ToxBank 2014), which is the proximal toxicant.

Category II.IV: Organophosphorus pesticides. Pesticides are another group of substances where information on active metabolites often is available. Organophosphorous pesticides are an example of another P450-mediated activating group of molecules. In this case, the primary toxicity is neurotoxicity originating from acetylcholinesterase (AChE) inhibition leading to increased neurotransmitter activity (Fukuto 1990). This category will not be followed up in the read-across case study because liver toxicity was prioritized within SEURAT-1.

*Scenario III: Chemicals with general low or no toxicity.* Category III.I: Propylene glycol ethers. Propylene glycol ethers are thought to exert no or very low toxicity and are used in cosmetic products. There is some evidence *in vivo* for general toxic effects such as increased liver and kidney weight (U.S. EPA 2010). These liver and kidney effects might be attributable to liver enzyme induction and accumulation of α_2u_-globulin, respectively, and are presumably induced by the parent compounds, although this has not been thoroughly investigated (Doi et al. 2007). In contrast, the ethylene glycol ethers cause both hematopoietic and reproductive effects (Wess 1992). Ethylene glycol ethers are mediated by alkoxyacid metabolites whose formation is catalyzed by ALDH/ADH (aldehyde dehydrogenase/alcohol dehydrogenase) (Lockley et al. 2005).

There is a considerable number of *in vitro* data from ToxCast (U.S. EPA 2014) for the propylene glycol ethers that confirm a general low toxicity profile. However, several of the analogues lack data, and a subset of the propylene glycol ethers with a more complete data set should be identified for the case study. One of the ethylene glycol ethers is suggested to be included in the category to better understand the difference in toxicity and to assist in gaining confidence in readout result from the assumed nontoxic substances.

We suggested that this category should be followed-up by external collaborative partners to SEURAT-1. The final outcome will be included in the case study.

Category III.II: Saturated alcohols. The saturated alcohols are another suitable example chosen based on available low repeated dose toxicity data for some of the long-chain compounds. In this case, the category has no obvious chemical reactivity, no obvious bioactivity, and high no observable effect doses (e.g., ≥ 500 mg/kg/day) for repeated-dose tested analogues (U.S. EPA 2010). Data from 28-day repeated-dose studies exist for several of these substances, and we suggest that available data can be read-across to category members without data. There are also ToxCast data (U.S. EPA 2014) for more than a dozen longer-chain (≥ C6) saturated alcohols. Only 0.1–0.3% of the ToxCast assays show activity for these alcohols, and then only at the highest concentration tested. In addition, none of the assays with positive outcome is associated to a specific bioactivity.

**Figure 3:**
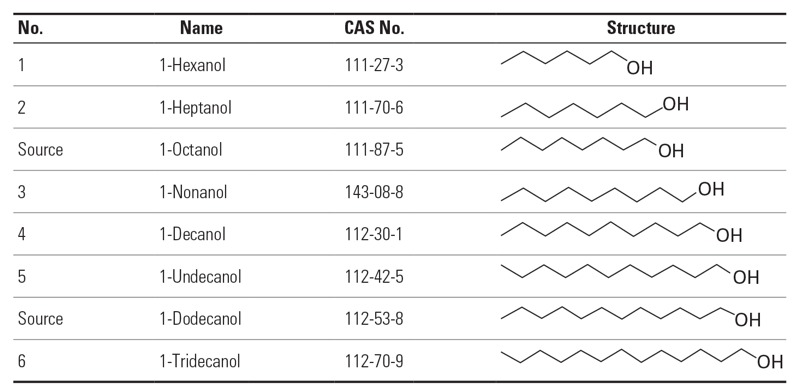
Chemicals suggested for Category III.II: saturated alcohols, identified by names, CAS numbers, and molecular structures. The two substances indicated “source” are the most data-rich substances in the category with known MoA.

We agreed that this category will be further investigated within the SEURAT-1 case study. The hypothesis is that saturated alcohols of intermediate chain length (i.e., C6–C13) are direct-acting toxicants with a similar reversible MoA leading to very low toxicity. The chemical category (Figure 3) is chosen based on carbon chain length. Repeated dose data for the primary source chemicals 1-octanol and 1-dodecanol can be read-across to untested saturated alcohols of similar chain lengths. The step 2 read-across exercise will be carried out based on additional *in vitro* evidence retrieved from ToxCast together with data from SEURAT-1 *in silico* methods.

*Scenario IV: Distinguishing chemicals in a structurally similar category with variable toxicities based on the MoA hypothesis.* The categories chosen for this scenario include chemicals with moderate toxicity, to distinguish them from scenario III, which includes chemicals with low and no toxicity. However, more emphasis is given to the specificity (i.e., MoA) rather than the potency of the toxic effect.

Category IV.I: Short-chain carboxylic acids (SCCAs). An advantage when selecting the SCCAs as a category is that the SEURAT-1 gold compound (ToxBank 2014; Wiseman 2012) valproic acid (VPA) can be used as a source for read across to other carboxylic acids. VPA is a branched-chain fatty acid that is recognized as a substrate by the fatty acid oxidation pathway causing steatosis through mitochondrial toxicity (ToxBank 2014). At least one of the target compounds selected should be a short-chained carboxylic acid with alkyl branching with high similarity to VPA. This compound is assumed to have the same MoA as VPA, and the observed effects would be similar; only the potency could vary. In addition the category should include carboxylic acids that are structurally less similar to VPA; for these, the variation in toxicity originates in different MoAs.

**Figure 4:**
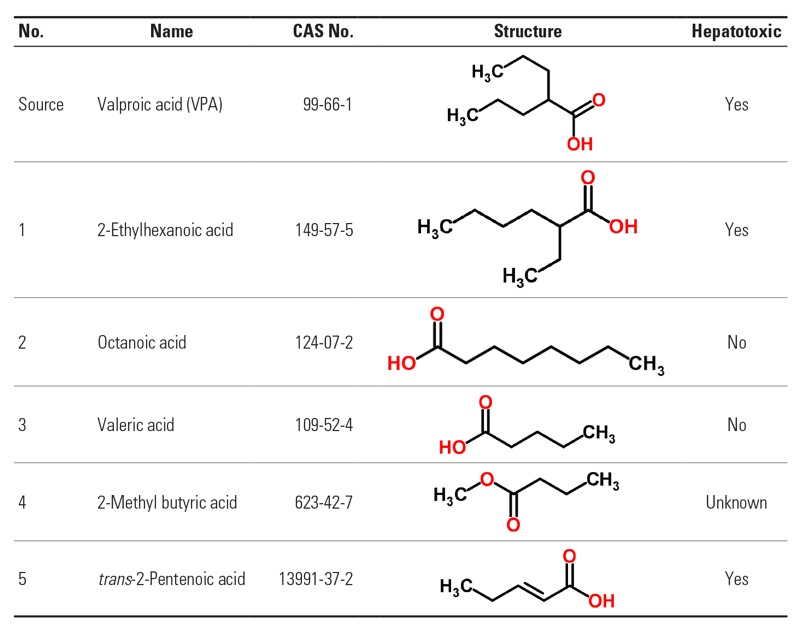
Chemicals suggested for Category IV.I: short-chain carboxylic acids (SCCAs), identified by names, CAS numbers, and molecular structures. The substance indicated “source” is the most data-rich substance in the category with known MoA.

The category will be further investigated within the SEURAT-1 read-across study. *In vitro* data will be collected from several SEURAT-1 assays. Additional testing for the selected chemicals will be performed and the resulting data will be included in the step-2 read-across. In addition, predictive computational models and profilers developed within the SEURAT-1 project COSMOS will contribute with additional data. Suggested chemicals to include in this VPA category are listed in Figure 4, including three negative targets and one positive. In addition, one unknown compound with an additional ester group was selected. This compound would be difficult to predict through solely the step 1 analysis. The additional *in vitro* data in the step 2 analysis should be able to show whether it reacts as VPA or with an alternative MoA, due to, for example, ester hydrolysis.

Category IV.II: Alkyl phenols. Another category suggested for this scenario is the alkyl phenols, which includes both different chain lengths and different branching. This category contains toxic or low-toxic chemicals. There are sufficient *in vivo* data (U.S. EPA 2010) available and there are also *in vitro* data from ToxCast (U.S. EPA 2014) for several of the chemicals in this category. The alkyl phenols are considered to be further investigated and included in the SEURAT-1 read-across case study.

## Concluding Remarks

When selecting read-across source and target compounds, it is necessary to address the following considerations:

Type of a read-across approach (analogue or category)
Characterization of the read-across scenario
Identification of the MoA of source compound
Read-across hypothesis
Similarity between source and target compounds
Whether human data are available
Whether *in vivo* animal data are available
Whether *in vitro* data are available
Uncertainties associated with the read-across both before and after the addition of the novel *in vitro* data.

Chemical categories were identified for four different decision contexts chosen to cover realistically possible read-across situations, as listed in Table 1. For each scenario, one category was selected to be included in the SEURAT-1 read-across case study. Each of the four selected read-across categories will now be investigated in a two-step procedure. In a first step, all available information (e.g., human and animal *in vivo* data, *in vitro* data, metabolism data) will be collected, including additional molecular descriptors to further investigate chemical similarities within a category. Read-across assessments will be conducted using available *in vivo* test data as their primary basis; in a second step, novel *in vitro* and *in silico* data will be integrated and the read-across assessment repeated. It will be necessary to evaluate robustness/confidence in the read-across assessment before and after the addition of the alternative data. It is also important to agree on how best to visualize results, especially from the novel data streams.

**Table 1:** Suggested read-across scenarios, corresponding chemical categories, and main adverse effects.

Read-across scenario	Chemical category	Main adverse effects
I. Chemical similarity of compounds that do not require (or do not undergo) metabolism to exert a potential adverse human health effect	I.I: Perfluoroalkyl acids (PFAAs)	Hepatotoxicity, developmental toxicity
	I.II: Phthalates	Reproductive toxicity, carcinogenicity
	I.III: Pesticides	Dependent on category of pesticides chosen
II. Chemical similarity involving metabolism (resulting in exposure to the same/similar toxicant)	II.I: β-Unsaturated alcohols (allylic alcohols)	Hepatotoxicity (fibrosis), kidney and lung toxicity
	II.II: Halogenated solvents	Liver and kidney toxicity
	II.III: Pharmaceuticals, such as acidic drugs or acetaminophen (Tylenol^®^, Paracetamol^®^) with analogues	Mainly hepatotoxicity concern
	II.IV: Organophosphorus pesticides	Neurotoxicity
III. Chemicals with general low or no toxicity	III.I: Propylene glycol ethers	Low/no toxicity
	III.II: Primary alcohols	Low/no toxicity
IV. Distinguishing chemicals in a structurally similar category with variable toxicities based on the MoA hypothesis	IV.I: Short-chain carboxylic acids (SCCAs)	Hepatotoxicity (steatosis)
	IV.II: Alkyl phenols	Hepatotoxicity and reproductive toxicity for certain members of the category

The aim of the exercise described herein is to strengthen the read-across arguments with *in vitro* and *in silico* profiling and toxicokinetics modeling for the four read-across scenarios with illustrative examples of chemical groups for each decision context. Scenario III is especially challenging, including the read-across of no or low toxicity. How to address uncertainty when reading across no adversity was discussed in a recent published case study (Ball et al. 2014) and will be further considered in the present exercise.

Based on the outcomes of the workshop and follow-up studies, an advisory document will be drafted on how to apply data from alternative methods to support read-across categories, with the intention to propose the document as an accompaniment to the new OECD guidance on chemical grouping (OECD 2014a). The suggested SEURAT-1 read-across methodology could also be linked with the more formalized approach already implemented into the OECD QSAR Toolbox.

## References

[r1] Ankley GT, Bennett RS, Erickson RJ, Hoff DJ, Hornung MW, Johnson RD (2010). Adverse outcome pathways: a conceptual framework to support ecotoxicology research and risk assessment.. Environ Toxicol Chem.

[r2] Ball N, Bartels M, Budinsky R, Klapacz J, Hays S, Kirman C (2014). The challenge of using read-across within the EU REACH regulatory framework; how much uncertainty is too much? Dipropylene glycol methyl ether acetate, an exemplary case study.. Regul Toxicol Pharmacol.

[r3] Berggren E. (2014). SEURAT-1 proof-of-concepts. In: Towards the Replacement of *in Vivo* Repeated Dose Systemic Toxicity Testing. Vol. 4 (Gocht T, Schwarz M, eds). Le Perreux-sur-Marne, France:Coach Consortium, 86–93.. http://www.seurat-1.eu/media/download_gallery/SEURAT-1_Annual%20Report_Vol%204_HD.pdf.

[r4] Blackburn K, Bjerke D, Daston G, Felter S, Mahony C, Naciff J (2011). Case studies to test: a framework for using structural, reactivity, metabolic and physicochemical similarity to evaluate the suitability of analogs for SAR-based toxicological assessments.. Regul Toxicol Pharmacol.

[r5] Blackburn K, Stuard SB (2014). A framework to facilitate consistent characterization of read across uncertainty.. Regul Toxicol Pharmacol.

[r6] Boobis AR, Doe JE, Heinrich-Hirsch B, Meek ME, Munn S, Ruchirawat M (2008). IPCS framework for analyzing the relevance of a noncancer mode of action for humans.. Crit Rev Toxicol.

[r7] Bradbury SP, Christensen GM (1991). Inhibition of alcohol dehydrogenase activity by acetylenic and allylic alcohols: concordance with in vivo electrophilic reactivity in fish.. Environ Toxicol Chem.

[r8] Cosmetics Europe. (2015). Cosmetics Europe, the Personal Care Association Homepage.. https://www.cosmeticseurope.eu/.

[r9] COSMOS (Cosmetics to Optimise Safety). (2011). The SEURAT-1 Project COSMOS Homepage.. http://www.cosmostox.eu/home/welcome/.

[r10] COSMOS. (2014). The COSMOS Database on Chemical and Toxicological Data for Cosmetics Ingredients.. http://cosmosdb.cosmostox.eu/.

[r11] Cronin MTD, Madden JC, Enoch SJ, Roberts DW. (2013). Chemical Toxicity Prediction: Category Formation and Read-Across.

[r12] Davis CD, Hanumegowda UM. (2009). The role of drug metabolism in toxicology. In: Drug Metabolism Handbook: Concepts and Applications (Nassar AF, Hollenberg PF, Scatina J, eds).

[r13] Doi AM, Hill G, Seely J, Hailey JR, Kissling G, Bucher JR (2007). α2u-Globulin nephropathy and renal tumors in national toxicology program studies.. Toxicol Pathol.

[r14] EC (European Commission). (2004). Trichloroethylene. CAS No: 79-01-6. EINECS No: 201-167-4. Summary Risk Assessment Report.. http://echa.europa.eu/documents/10162/d30e53cc-89e7-4d1c-89c0-7ec216f84d48.

[r15] EC. (2005). Tetrachloroethylene: Part I – Environment. CAS No: 127-18-4. EINECS No: 204-825-9. Summary Risk Assessment Report.. http://echa.europa.eu/documents/10162/733515ca-7d61-463c-9cde-af560097ce25.

[r16] EC. (2007). Research and Innovation: FP7 Homepage.. http://ec.europa.eu/research/fp7/.

[r17] EC. (2013). Scientific Committee on Consumer Safety (SCCS) Homepage.. http://ec.europa.eu/health/scientific_committees/consumer_safety/.

[r18] ECHA (European Chemicals Agency). (2008). Guidance on Information Requirements and Chemical Safety Assessment. Chapter R.6: QSARs and Grouping of Chemicals.. http://echa.europa.eu/documents/10162/13632/information_requirements_r6_en.pdf.

[r19] ECHA. (2012). Practical Guide 6. How to Report Read-Across and Categories. ECHA-10-B-11.1-EN.. http://echa.europa.eu/documents/10162/13655/pg_report_readacross_en.pdf.

[r20] ECHA. (2013a). Grouping of Substances and Read-Across Approach. Part 1: Introductory Note. ECHA-13-R-02-EN.. http://echa.europa.eu/documents/10162/13628/read_across_introductory_note_en.pdf.

[r21] ECHA. (2013b). Read-Across Illustrative Example. Part 2: Example 1 – Analogue Approach: Similarity Based on Breakdown Products. ECHA-13-R-03-EN.. http://echa.europa.eu/documents/10162/13628/read_across_example_1_en.pdf.

[r22] ECHA. (2014). The Use of Alternatives to Testing on Animals for the REACH Regulation. Second Report under Article 117(3) of the REACH Regulation. ECHA-14-A-07-EN.. http://echa.europa.eu/documents/10162/13639/alternatives_test_animals_2014_en.pdf.

[r23] EFSA (European Food Safety Authority). (2015). European Food Safety Authority Homepage.. http://www.efsa.europa.eu/.

[r24] EU (European Union). (2006). Regulation (EC) No 1907/2006 of the European Parliament and of the Council of 18 December 2006 concerning the Registration, Evaluation, Authorisation and Restriction of Chemicals (REACH).. http://eur-lex.europa.eu/legal-content/EN/TXT/?qid=1428942204927&uri=CELEX:32006R1907.

[r25] EU. (2009). Regulation (EC) No 1223/2009 of the European Parliament and of the Council of 30 November 2009 on Cosmetic Products.. http://eur-lex.europa.eu/legal-content/EN/TXT/?qid=1428942330235&uri=CELEX:32009R1223.

[r26] EU. (2010). Directive 2010/63/EU of the European Parliament and of the Council of 22 September 2010 on the Protection of Animals Used for Scientific Purposes.. http://eur-lex.europa.eu/legal-content/EN/TXT/?qid=1428942397721&uri=CELEX:32010L0063.

[r27] Fukuto TR (1990). Mechanism of action of organophosphorus and carbamate insecticides.. Environ Health Perspect.

[r28] HeMiBio (Hepatic Microfluidic Bioreactor). (2011). The SEURAT-1 Project HeMiBio Homepage.. http://www.hemibio.eu/.

[r29] Johnson AM, Maggiora GM. (1990). Concepts and applications of Molecular Similarity..

[r30] Kleinstreuer NC, Yang J, Berg EL, Knudsen TB, Richard AM, Martin MT (2014). Phenotypic screening of the ToxCast chemical library to classify toxic and therapeutic mechanisms.. Nat Biotechnol.

[r31] Krewski D, Acosta D, Andersen M, Anderson H, Bailar JC, Boekelheide K (2010). Toxicity testing in the 21st century: a vision and a strategy.. J Toxicol Environ Health B Crit Rev.

[r32] Landesmann B, Goumenou M, Munn S, Whelan M. (2012). Description of Prototype Modes-of-Action Related to Repeated Dose Toxicity. European Commission, Joint Research Centre, JRC Scientific and Policy Report 75689.. http://publications.jrc.ec.europa.eu/repository/handle/JRC75689.

[r33] Landesmann B, Vinken M. (2013). Mode-of-Action Working Group: capturing mode-of-action knowledge. In: Towards the Replacement of *in Vivo* Repeated Dose Systemic Toxicity Testing. Vol. 3 (Gocht T, Schwarz M, eds). Le Perreux-sur-Marne, France:Coach Consortium, 283–321.. http://www.seurat-1.eu/media/download_gallery/SEURAT%201-v3.pdf.

[r34] Lockley DJ, Howes D, Williams FM (2005). Cutaneous metabolism of glycol ethers.. Arch Toxicol.

[r35] Lostia A. (2014). Mode-of-action-based classification model for repeated dose liver toxicity. In: Towards the Replacement of *in Vivo* Repeated Dose Systemic Toxicity Testing. Vol. 4 (Gocht T, Schwarz M, eds). Le Perreux-sur-Marne, France:Coach Consortium, 104–106.. http://www.seurat-1.eu/media/download_gallery/SEURAT-1_Annual%20Report_Vol%204_HD.pdf.

[r36] Low Y, Sedykh A, Fourches D, Golbraikh A, Whelan M, Rusyn I (2013). Integrative chemical–biological read-across approach for chemical hazard classification.. Chem Res Toxicol.

[r37] National Research Council. (2007). Toxicity Testing in the 21st Century: a Vision and a Strategy. Washington, DC:National Academies Press.. http://www.nap.edu/openbook.php?record_id=11970.

[r38] Nelson JW Hatch EE Webster TF 2010 Exposure to polyfluoroalkyl chemicals and cholesterol, body weight, and insulin resistance in the general U.S. population. Environ Health Perspect 118 197 202; 10.1289/ehp.0901165 20123614 PMC2831917

[r39] Noor F, Heinzle E. (2014). Evaluation of valproic acid induced steatosis in HepaRG cells. In: Towards the Replacement of *in Vivo* Repeated Dose Systemic Toxicity Testing. Vol. 4 (Gocht T, Schwarz M, eds). Le Perreux-sur-Marne, France:Coach Consortium, 98–99.. http://www.seurat-1.eu/media/download_gallery/SEURAT-1_Annual%20Report_Vol%204_HD.pdf.

[r40] OECD (Organisation for Economic Co-operation and Development). (2013a). Guidance Document on Developing and Assessing Adverse Outcome Pathways. ENV/JM/MONO(2013)6.. http://www.oecd.org/officialdocuments/publicdisplaydocumentpdf/?cote=env/jm/mono%282013%296&doclanguage=en.

[r41] OECD. (2013b). QSAR Toolbox Homepage. The OECD QSAR Toolbox for Grouping Chemicals into Categories.. http://www.qsartoolbox.org/home.

[r42] OECD. (2014a). Guidance on Grouping of Chemicals, Second Edition. ENV/JM/MONO(2014)4.. http://www.oecd.org/officialdocuments/publicdisplaydocumentpdf/?cote=env/jm/mono%282014%294&doclanguage=en.

[r43] OECD. (2014b.). The Adverse Outcome Pathway Knowledge Base (AOP-KB): AOP-Wiki.. https://aopkb.org.

[r44] O’Shea SH, Schwarz J, Kosyk O, Ross PK, Ha MJ, Wright FA (2011). *In vitro* screening for population variability in chemical toxicity.. Toxicol Sci.

[r45] Reif DM Martin MT Tan S Houck KA Judson R Richard AM 2010 Endocrine profiling and prioritization of environmental chemicals using ToxCast data. Environ Health Perspect 118 1714 1720; 10.1289/ehp.1002180 20826373 PMC3002190

[r46] Reif DM, Sypa M, Lock EF, Wright FA, Wilson A, Cathey T (2013). ToxPi GUI: an interactive visualization tool for transparent integration of data from diverse sources of evidence.. Bioinformatics.

[r47] RIFM (Research Institute for Fragrance Materials). (2014). The RIFM Database.. http://www.rifm.org/rifm-science-database.php.

[r48] Rogers DJ, Tanimoto TT (1960). A computer program for classifying plants.. Science.

[r49] Rusyn I, Corton JC (2012). Mechanistic considerations for human relevance of cancer hazard of di(2-ethylhexyl) phthalate.. Mutat Res.

[r50] Schultz TW Amcoff P Berggren E Gautier F, Klaric, Knight DJ, et al. 2015 A strategy for structuring and reporting a read-across prediction of toxicity. Regul Toxicol Pharmacol 72 586 601 26003513 10.1016/j.yrtph.2015.05.016

[r51] SEURAT-1 (Safety Evaluation Ultimately Replacing Animal Testing, Step 1). (2011). SEURAT-1 Homepage.. http://www.seurat-1.eu/.

[r52] Strubelt O, Deters M, Pentz R, Siegers CP, Younes M (1999). The toxic and metabolic effects of 23 aliphatic alcohols in the isolated perfused rat liver.. Toxicol Sci.

[r53] Sturla SJ, Boobis AR, FitzGerald RE, Hoeng J, Kavlock KJ, Schirmer K (2014). Systems toxicology: from basic research to risk assessment.. Chem Res Toxicol.

[r54] Todeschini R, Consonni V. (2009). Molecular Descriptors for Chemoinformatics. 2nd ed.

[r55] ToxBank. (2014). Gold Compounds Wiki Pages.. http://www.toxbank.net/compound-wiki.

[r56] U.S. ATSDR (U.S. Agency for Toxic Substances and Disease Registry). (2002). Toxicological Profile for Di(2-ethylhexyl)phthalate (DEHP).. http://www.atsdr.cdc.gov/ToxProfiles/tp.asp?id=684&tid=65.

[r57] U.S. ATSDR. (2014a). Toxicological Profile for Tetrachloroethylene (PERK). Draft for Public Comment.. http://www.atsdr.cdc.gov/ToxProfiles/tp.asp?id=265&tid=48.

[r58] U.S. ATSDR. (2014b). Toxicological Profile for Trichloroethylene (TCE). Draft for Public Comment.. http://www.atsdr.cdc.gov/ToxProfiles/tp.asp?id=173&tid=30.

[r59] U.S. EPA (U.S. Environmental Protection Agency). (2010). ToxRefDB [Toxicity Reference Database].. http://actor.epa.gov/toxrefdb.

[r60] U.S. EPA. (2014). ToxCast™ Data.. http://www.epa.gov/ncct/toxcast/data.html.

[r61] van Leeuwen K, Schultz TW, Henry T, Diderich R, Veith GD (2009). Using chemical categories to fill data gaps in hazard assessment.. SAR QSAR Environ Res.

[r62] Wambaugh JF, Setzer RW, Pitruzzello AM, Liu J, Reif DM, Kleinstreuer NC (2013). Dosimetric anchoring of *in vivo* and *in vitro* studies for perfluorooctanoate and perfluorooctanesulfonate.. Toxicol Sci.

[r63] Wess AJ (1992). Reproductive toxicity of ethylene glycol monomethyl ether, ethylene glycol monoethyl and their acetates.. Scand J Work Environ Health.

[r64] Wetmore BA, Wambaugh JF, Ferguson SS, Sochaski MA, Rotroff DM, Freeman K (2012). Integration of dosimetry, exposure, and high-throughput screening data in chemical toxicity assessment.. Toxicol Sci.

[r65] Whelan M, Schwarz M, Scientific Expert Panel of the SEURAT-1 Research Initiative. (2011). SEURAT: vision, research strategy and execution. In: Towards the Replacement of *in Vivo* Repeated Dose Systemic Toxicity Testing. Vol. 1 (Gocht T, Schwarz M, eds). Le Perreux-sur-Marne, France:Coach Consortium, 47–52.. http://www.seurat-1.eu/media/download_gallery/SEURAT-1_Annual%20Report_Vol%201_Sept2011_HR.pdf.

[r66] Whelan M, Schwarz M, Scientific Expert Panel of the SEURAT-1 Research Initiative. (2012). Elaborating the SEURAT-1 research strategy. In: Towards the Replacement of *in Vivo* Repeated Dose Systemic Toxicity Testing. Vol. 2 (Gocht T, Schwarz M, eds). Le Perreux-sur-Marne, France:Coach Consortium, 47–63.. http://www.seurat-1.eu/media/download_gallery/SEURAT%201v2.

[r67] Whelan M, Schwarz M, Scientific Expert Panel of the SEURAT-1 Research Initiative. (2013). The SEURAT-1 research strategy: proving concepts. In: Towards the Replacement of *in Vivo* Repeated Dose Systemic Toxicity Testing. Vol. 3 (Gocht T, Schwarz M, eds). Le Perreux-sur-Marne, France:Coach Consortium, 57–72.. http://www.seurat-1.eu/media/download_gallery/SEURAT%201-v3.pdf.

[r68] White A, Knight D. (2014). Safety Assessment Working Group. In: Towards the Replacement of *in Vivo* Repeated Dose Systemic Toxicity Testing. Vol. 4 (Gocht T, Schwarz M, eds). Le Perreux-sur-Marne, France:Coach Consortium, 327–331.. http://www.seurat-1.eu/media/download_gallery/SEURAT-1_Annual%20Report_Vol%204_HD.pdf.

[r69] Wilkening S, Stahl F, Bader A (2003). Comparison of primary human hepatocytes and hepatoma cell line HepG2 with regard to their biotransformation properties.. Drug Metab Dispos.

[r70] Wiseman J. (2012). Mechanism-based selection of reference compounds for toxicity testing procedures. In: Towards the Replacement of *in Vivo* Repeated Dose Systemic Toxicity Testing. Vol. 2 (Gocht T, Schwarz M, eds). Le Perreux-sur-Marne, France:Coach Consortium, 278–281.. http://www.seurat-1.eu/media/download_gallery/SEURAT%201v2.

[r71] Wu S, Blackburn K, Amburgey J, Jaworska J, Federle T (2010). A framework for using structural, reactivity, metabolic and physicochemical similarity to evaluate the suitability of analogs for SAR-based toxicological assessments.. Regul Toxicol Pharmacol.

[r72] Zhang L, Ren XM, Guo LH (2013). Structure-based investigation on the interaction of perfluorinated compounds with human liver fatty acid binding protein.. Environ Sci Technol.

